# Solid Self-Nano Emulsifying Nanoplatform Loaded with Tamoxifen and Resveratrol for Treatment of Breast Cancer

**DOI:** 10.3390/pharmaceutics14071486

**Published:** 2022-07-18

**Authors:** Nupur Shrivastava, Ankit Parikh, Rikeshwer Prasad Dewangan, Largee Biswas, Anita Kamra Verma, Saurabh Mittal, Javed Ali, Sanjay Garg, Sanjula Baboota

**Affiliations:** 1 Department of Pharmaceutics, School of Pharmaceutical Education and Research, Jamia Hamdard, New Delhi 110062, India; nupurs.211@gmail.com (N.S.); saurabhmittal904@gmail.com (S.M.); jali@jamiahamdard.ac.in (J.A.); 2 Pharmaceutical Innovation and Development (PIDG) Group, Clinical and Health Sciences, University of South Australia, Adelaide, SA 5000, Australia; ankit.parikh@unisa.edu.au; 3 Department of Pharmaceutical Chemistry, School of Pharmaceutical Education and Research, Jamia Hamdard, New Delhi 110062, India; rpdewangan@jamiahamdard.ac.in; 4 Nano Biotech Lab, Department of Zoology, Kirori Mal College, University of Delhi, New Delhi 110007, India; largeebiswas@gmail.com (L.B.); akamra23@hotmail.com (A.K.V.)

**Keywords:** combination therapy, tamoxifen, resveratrol, solid self-nanoemulsifying drug delivery system, breast cancer, enhanced bioavailability, synergistic action

## Abstract

The solid self-nanoemulsifying drug delivery system (s-SNEDDS) is a growing platform for the delivery of drugs via oral route. In the present work, tamoxifen (TAM) was loaded in SNEDDS with resveratrol (RES), which is a potent chemotherapeutic, antioxidant, anti-inflammatory and P-gp inhibitor for enhancing bioavailability and to obtain synergistic anti-cancer effect against breast cancer. SNEDDS were developed using capmul MCM as oil, Tween 80 as surfactant and transcutol-HP as co-surfactant and optimized by central composite rotatable design. Neusilin US2 concentration was optimized for adsorption of liquid SNEDDS to prepare s-SNEDDS. The developed formulation was characterized and investigated for various in vitro and cell line comparative studies. Optimized TAM-RES-s-SNEDDS showed spherical droplets of a size less than 200 nm. In all in vitro studies, TAM-RES-s-SNEDDS showed significantly improved (*p* ˂ 0.05) release and permeation across the dialysis membrane and intestinal lumen. Moreover, TAM-RES-s-SNEDDS possessed significantly greater therapeutic efficacy (*p* < 0.05) and better internalization on the MCF-7 cell line as compared to the conventional formulation. Additionally, oral bioavailability of TAM from SNEDDS was 1.63 folds significantly higher (*p* < 0.05) than that of combination suspension and 4.16 folds significantly higher (*p* < 0.05) than TAM suspension. Thus, findings suggest that TAM- RES-s-SNEDDS can be the future delivery system that potentially delivers both drugs to cancer cells for better treatment.

## 1. Introduction

Breast cancer is a drastically increasing neoplastic disease, and it has become the most prevalent cancer worldwide. The United States and several Asian countries are extensively affected by this disease [1]. More than 1,500,000 Indian females are diagnosed with breast cancer every year [2]. In 2020, breast cancer contributed to 39.4% of total cancer found in India [3]. Globally, more than 2.3 million females are diagnosed with it and approximately 685,000 females die of it [4]. Estrogen is responsible for secondary sexual characteristics and maintaining the ideal uterine cycle in females. It has the intrinsic ability to initiate protein synthesis and proliferation that is responsible for the growth and development of the mammary gland [5]. There are two different hypotheses for estrogen-induced breast cancer. One is the receptor-oriented cancerous pathway where estrogen acts via estrogen-receptor alpha (ERα) and stimulates the proliferation of cells and initiates mutations, which turn out as a function of errors during DNA replication and leads to breast cancer [6]. Another one is the non-receptor-oriented cancerous pathway where metabolites of estrogen lead to the mutation of DNA and finally result in the development of breast cancer [7,8,9].

Tamoxifen (TAM) is considered as the ‘gold standard’ for breast cancer treatment and is valued as a lifesaving drug. It belongs to the category of selective estrogen receptor modulators (SERMs) widely used for the treatment of estrogen receptor positive breast cancer, which inhibits the interaction between estrogen and estrogen receptors [10]. Despite the beneficial therapeutic uses of this drug, there are many other less favorable properties including low aqueous solubility (0.04 µg/mL), substrate to p-glycoprotein efflux in the gastrointestinal membrane leading to its removal from the absorption site and high first-pass metabolism in liver finally limiting the bioavailability [11,12]. Long term oral administration of TAM leads to the development of resistance against TAM, which ultimately affects the efficacy of the drug [13]. Hence, to overcome these problems, in the present study, the combination approach was used where TAM was combined with resveratrol (RES) in single drug delivery.

This combination acts on both the hypothesis, i.e., TAM blocks the receptor-oriented cancerous pathway, whereas RES due to its free-radical scavenging property hinders the non- receptor-oriented cancerous pathway. RES is a natural non-flavonoid polyphenol stilbene with anticancer, anti-aging, anti-oxidant, anti-inflammatory, blood sugar lowering activity and beneficial cardiovascular effects [14,15]. RES can also block the transformation of procarcinogen to carcinogen [16]. The molecular mechanism and signalling pathways proposed for its anticancer effect includes the modulation of epidermal growth factor receptor (EGF-R), human epidermal growth factor receptor- 2 (HER-2), vascular endothelial growth factor (VGEF) and extracellular signal-regulated kinase (ERK) activity where it prevents the initiation, progression and suppression of carcinogenesis [17]; alteration in the protein kinase G (PKG)/cyclic guanosine monophosphate (cGMP)/inhibitor of apoptosis protein (IAP) activity that results in cytotoxicity; alteration in the phosphoinositide- 3-kinase (PI3K)/protein kinase B (AKT)/mammalian target of rapamycin (mTOR)/mitogen-activated protein kinases (MAPKs) signalling pathway where it reduces the formation of multi-protein-like receptor protein tyrosine kinases (RPTKs) and its ability to increase the activities of caspase 3,9; and apoptosis-inducing factor (AIF) that results in a direct cytotoxic effect on cancerous cells [14]. Apart from these proposed mechanistic pathways of resveratrol-induced anticancer effect, it also alters the cytoplasmic tyrosine kinase signalling pathways, such as the NF-kB essential modulator (NEMO)/tumor necrosis factor-α (TNF-α) pathway and sirutin (SIRT)/p53 pathway [18]. Furthermore, RES acts as an inhibitor of the p-glycoprotein efflux pump in the gastrointestinal membrane and augments the bioavailability of anti-cancer drugs at the action site. Overall, it synergizes the therapeutic efficacy of other anti-cancer agents such as TAM by scavenging the free radicals and inhibiting cell proliferation, the P-gp efflux pump and various enzymes, including ribonucleotide reductase, DNA polymerases and protein kinase C (PKC). As well as its valuable activity, RES has various undesirable properties, such as poor bioavailability, low water solubility (0.05 mg/mL) and chemical instability [19]. Previous studies by Chowdhary et al. have reported the solid dispersion of a combination of TAM and RES for enhanced bioavailability but did not mention any studies to prove the synergistic anti-cancer action against breast cancer [20]. Another study by Shi et al., reported that RES can sensitize the tamoxifen-resistant cells to tamoxifen but did not prepare any drug loaded into a nano carrier [21].

Despite the anti-cancer action of both drugs, the biopharmaceutical issues associated with this combination are poor solubility as both belong to BCS class II, poor bioavailability and intrinsic toxicity. Thus, to attain the significant therapeutic benefits from a combination of TAM and RES in a single carrier, smart drug delivery must be approached. There are many techniques to enhance the solubility of drugs such as size reduction, polymorphism, solid dispersion in polymer, complexation and prodrugs, which can overcome the release rate challenges but are not able to deal with gastric degradation-related bioavailability challenges [22,23].

In recent years, the foremost favorable lipid-based drug delivery system which can overcome the above-mentioned challenges is a self-nanoemulsifying drug delivery system (SNEDDS). This is the most popular, commercially acceptable drug delivery to enhance the bioavailability of orally administrable drugs [22]. In comparison to other lipid-based formulations, SNEDDS provide a larger interfacial area for the partitioning of drugs between oil and water, and ultimately offer easy dispersibility [24]. The SNEDDS is a blend of oil, surfactant and co-surfactant which forms small droplets of a size less than 200 nm of nanoemulsion when it comes into contact with the gastro-intestinal fluid (GIF) following oral administration [25]. The oily phase of SNEDDS is converted into intestinal micelles followed by a chain of conversion via enzymatic action of gastric lipase. These micelles can directly drain the encapsulated drug into the lymphatic system from where it can easily reach the targeted site, thereby avoiding the first pass metabolism and enhancing the oral bioavailability [26].

In spite of several benefits, the practical limitation of liquid SNEDDS such as the unusual interaction between the liquid and capsule shell, the possible precipitation of drugs and excipients at a lower temperature can be prevented by the solidification of liquid SNEDDS. Solid SNEDDS (s- SNEDDS) is the stable dosage form having high stability with ease of handling and can easily be taken by the patient [27].

The novelty of the current research work was related to the preparation of SNEDDS loaded with TAM and RES by systematically optimizing the concentration of oil, surfactant and co-surfactant. After that, the liquid SNEDDS was adsorbed over a solid carrier, i.e., Neusilin US2 and converted into s-SNEDDS for better stability and storage. Then, the developed formulation was extensively characterized for droplet size, percentage of transmittance, zeta potential, robustness to dilutions, in vitro release and different stability studies to confirm the retainability and quality under different physiological conditions. Further, we testified the intestinal permeability, depth of internalization in the intestinal wall and pharmacokinetic parameter of the developed formulation. Finally, we also demonstrated the synergistic anti-cancer action of the developed formulation with the anti-oxidant effect of resveratrol along with the internalization of SNEDDS on MCF- 7, the breast cancer cell line.

## 2. Materials and Methods

### 2.1. Materials

Resveratrol was acquired as a gift sample from Sigma-Aldrich, Bangalore, India, whereas tamoxifen was also acquired as a gift sample from Bioxera Pharma Pvt. Ltd., Mumbai, India. Various other solvents and chemicals of analytical grade were used throughout this project. For all experiments, deionized water was used.

### 2.2. Determination of Combination Index (CI)

The combination index for TAM and RES was determined using human breast adenocarcinoma cells (MCF-7) obtained from the National Centre for Cell Science, Pune, India. Briefly, Dulbecco’s Modified Eagle’s Medium (DMEM) contained in a 96-well culture plate was seeded with MCF-7 cells at a density of 5 × 10^3^ cells/well. The plate was supplemented with fetal bovine serum (FBS) (10%,) penicillin (100 units/mL) and streptomycin (100 mg/mL), followed by incubation for 24 h at the temperature of 37 ± 2 °C with 5% CO_2_ in the atmosphere. Then, after attaining 90% confluency, the cells were used for further studies. Cells were subjected to different samples of TAM and RES combinations in various ratios such as 1:1; 1:5; 1:10 and incubated for 24 h at the temperature of 37 ± 2 °C [28,29].

Cell cytotoxicity was studied by MTT (3-(4,5-dimethylthiazol-2-yl)-2,5-diphenyltetrazolium bromide) tetrazolium reduction assay. Where yellow color MTT is added into the cells it changes into purple formazan crystals because of the presence of NAD(P)H-dependent oxidoreductase enzymes in viable cells. Thus, after diluting the formazan crystals with suitable media, we can easily quantify the viable cells and the effect of the anti-cancer drug over the cells by using an ELISA reader plate [30].

For this study, each well of the pre-treated plate were again treated with 20 µL of MTT solution (5 mg/mL in PBS pH 7.4) and incubated for 4 h at the temperature of 37 °C for the formation formazan crystals that were further dissolved in 150 µL of dimethyl sulphoxide (DMSO). Then, the ELISA reader plate (Synergy HT, Bio-Tek, Winooski, VT, USA) was used to measure the optical density at 570 nm. The percentage of cytotoxicity was calculated using Equation (1):

(1) $$\%\;\mathrm{Cytotoxicity}\;=\frac{\mathrm{Absorbance}\;\mathrm{of}\;\mathrm{the}\;\mathrm{control}-\mathrm{Absorbance}\;\mathrm{of}\;\mathrm{the}\;\mathrm{test}\;}{\mathrm{Absorbance}\;\mathrm{of}\;\mathrm{the}\;\mathrm{control}}\times 100$$

After cell cytotoxicity analysis, IC_50_ was calculated. For the evaluation of the type of association between two compounds, the combination index (CI) was determined using Equation (2):

(2) $$CI=\frac{D_{1}}{Dx_{1}}+\frac{D_{2}}{Dy_{2}}$$

where *D*_1_ and *D*_2_ are the IC_50_ doses of two drugs in combination and *Dx*_1_ and *Dy*_2_ are the IC_50_ doses of two drugs alone. *CI* less than 1 indicates synergy, *CI* equal to 1 is additive effect, and *CI* more than 1 indicates antagonism [20,28].

### 2.3. Selection of Excipients

#### 2.3.1. Selection of Oil

For the determination of the solubility of TAM and RES in selected oils, the shake flask method was adopted [31]. An excess amount of TAM and RES were separately added to 400 µL of different oils, namely captex 355, migloyl 829, captex 100, castor oil, capmul PG8NF, linseed oil, almond oil, wheat germ oil and capmul MCM EP, separately present in the glass vials, and vortexed for 5 min. Then, the glass vials were shaken for 72 h in an isothermal shaker bath maintained at 37 °C ± 2 °C [32]. After that, mixtures were centrifuged at 500 rpm for separation of the supernatant, which was collected and diluted with methanol for the estimation of TAM and RES by UV spectrophotometer at their maximum wavelength of 237 nm and 306 nm, respectively.

#### 2.3.2. Selection of Surfactants and Co- Surfactants

Surfactants were selected on the basis of their emulsification ability for the selected oil, for which 200 µL of the selected oil was mixed with 200 µL of different surfactants, such as labrafac lipophile WL 1349, caproyl PGMS, cremophore and Tween 80, separately and vortexed for 5 min and kept at temperature between 40 and 45 °C for 5 min [33]. Co- surfactants were then selected on the ability to form emulsion with the selected oil and surfactant. The resultant mixtures obtained were diluted with deionized warm water to obtain an emulsion [29]. The resultant emulsions were then allowed to stand for 2 h followed by measuring the percentage of transmittance by UV spectrophotometer at 510 nm.

#### 2.3.3. Construction of Pseudoternary Phase Diagram

For the SNEDDS development, a pseudoternary phase diagram was created to calculate the surfactant:co-surfactant (S_mix_) and oil: S_mix_ ratios. The S_mix_ were taken in the ratios of 1:0, 1:1, 2:1, 3:1, 4:1, 5:1, 1:2. Then the mixture of oil and S_mix_ in the ratios of 1:9, 2:8, 3:7, 4:6, 5:5, 6:4, 7:3, 8:2, 9:1, 1:2, 1:3, 1: 3.5, 1:5, 1:6, 1: 7, 1:8 were titrated against deionized water. The volume of deionized water was varied between 5 and 95% at an interval of 5%. After the addition of deionized water to the mixture, it was vortexed for approximately between 2 and 5 min and visually observed for transparency or turbidity and those observations were recorded on the phase diagram. The three vertices of the pseudoternary phase diagram represent the S_mix_, oil and deionized water phase of the formulation. The percentage composition of each nanoemulsion was marked as a point on the pseudoternary phase diagram and the area enclosed under these points was noted as a nanoemulsion region. For formulation development, a percent composition with the greatest nanoemulsion region was chosen. [34,35].

### 2.4. Application of Quality by Design (QbD)

**Figure 1:**
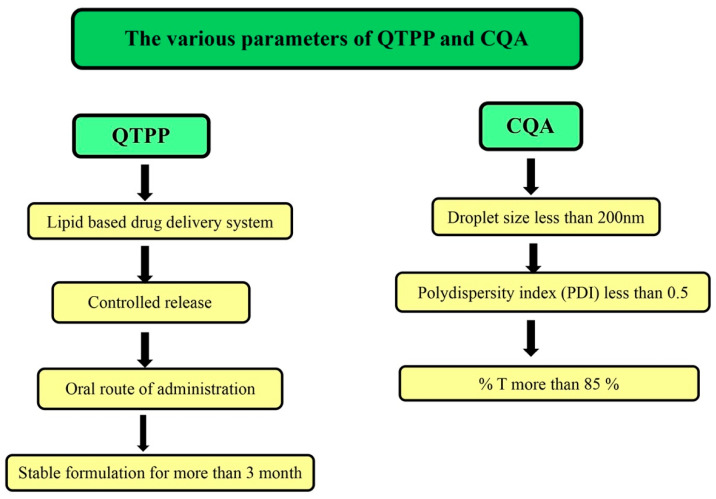
Parameters of QTPP and CQA for lipid-based drug delivery.

Quality by design adds value and quality to the final pharmaceutical product. It is necessary to identify and control the critical parameters related to the process and the product during the formulation development. This application provides a correlation between critical quality attributes (CQA), the quality target of product profile (QTPP), critical material attributes (CMA) and critical process parameters (CPP). The main agenda of using Qbd in the pharmaceutical industry is to develop a robust pharmaceutical product which can achieve the best therapeutic efficacy, quality attributes and long shelf life over storage. The various parameters of QTPP and CQA for the development of lipid-based drug delivery, i.e., SNEDDs, are given in Figure 1. The associated risk assessment profile was determined using the fish-bone diagram also known as an Ishikawa diagram (Minitab 17 software, M/s Minitab Inc., Philadelphia, PA, USA).

### 2.5. Formulation and Optimization of SNEDDS

The SNEDDS was optimized using response surface methodology (RSM), a design expert ^®^ software (version 10.0.4.1, State Ease Inc, Minneapolis, MN, USA). It is a statistical model widely utilized to investigate the infused effect of certain variables on experimental outcomes. It is also used to optimize the various parameters in the multivariable system. The CCRD was selected to study the effect of process variables on the expected responses. The combination of mathematical and statistical measures was embedded in the CCRD for modeling and analyzing the issues regarding the response of interest that comprise variables matrices. Further, it helps to gather the maximum information about the response of interest with a reduction in the number of trials. Thus, it simplifies the process of optimization by facilitating the determination of variable factors and the optimal range for the required response [36,37].

The independent factors chosen were concentration of oil and S_mix_ and their effect on droplet size; PDI and percentage of transmittance was observed (Table 1). CCRD generated thirteen runs with quadratic polynomial Equation (3), which described the relationship between the evaluated responses (Y) and independent variables (X_1_, X_2_) for the experiment, as shown in Equation (3):

Y = B0 + B_1_X_1_ + B_2_X_2_ + B_12_X_1_X_2_ + B_11_X_1_^2^ + B_22_X_2_^2^ (3)

### 2.6. Preparation of TAM and RES-Loaded Liquid SNEDDS

The precisely weighed TAM (10 mg) and RES (100 mg) in the ratio of 1:10 were dissolved in a mixture of 0.600 mL of oil and 1.860 mL of S_mix._ for the preparation of liquid SNEDDS. The resulting mixture was stirred using a magnetic stirrer with a speed of 100 rpm at 45 ± 2 °C for homogenization. The prepared liquid SNEDDS was then stored in glass vials and observed for phase separation and turbidity [13,38].

### 2.7. Conversion of SNEDDS into Solid SNEDDS

The solid carrier, neusilin US2, was used for the adsorption of optimized liquid SNEDDS into solid SNEDDS (s-SNEDDS). It is a synthetic carrier made up of amorphous magnesium aluminometasilicate. It is porous in nature and provides a large surface area for adsorption of large amounts of oils so that they can be incorporated into tablets. It has a very high adsorption capacity compared to other solid carriers [39]. The concentration of neusilin US2 (solid carrier) required for the solidification of the formulation was determined by adding increment amounts (100 mg) of it to the optimized liquid SNEDDS until the liquid was completely adsorbed and solidified properly.

### 2.8. Characterization of s-SNEDDS

#### 2.8.1. Droplet Size and PDI

The Zeta sizer (Malvern Instruments Ltd., Malvern, UK) was utilized to determine the droplet size and PDI. The instrument works on the principle of dynamic light scattering (DLS). s-SNEDDS of 100 mg were diluted with 90 mL of deionized water, sonicated for 50 s and left undisturbed for 2 h. Then, the evaluation of droplet size and PDI of the collected samples was performed at 25 ± 2 °C with a constant refractive index of 1.471 [40]. All the observations were carried out in triplicate.

#### 2.8.2. Percentage Transmittance

The percentage of transmittance is an important parameter to check the isotropic nature of the SNEDDS, where the value closer to 100 implied clear formulations with a droplet size within the nanometric range having a larger surface area for drug release, which ultimately results in enhanced oral bioavailability. A UV spectrophotometer was used to determine the percentage of transmittance of the s-SNEDDS. Then, 100 mg of s-SNEDDS were diluted with 90 mL of deionized water, sonicated for 50 s and left undisturbed for 2 h. Next, the percentage of transmittance of the samples were measured at 510 nm with deionized water as blank [40]. All the observations were carried out in triplicate.

#### 2.8.3. Zeta Potential

The zeta sizer was used to measure the zeta potential of the s-SNEDDS. Zeta potential measurement assessed the electrophoretic mobility of the nanoparticles of the s-SNEDDS dispersed in liquid [41]. s-SNEDDS were dispersed and diluted with deionized water for analysis. All the observations were carried out in triplicate.

#### 2.8.4. Robustness to Dilutions

The robustness to dilutions is used to check the formulation stability without any phase separation and drug precipitation in the lumen of the gut where it will come into contact with different digestive fluids in various volumes. This study gave an indication about the suitability of the formulation for oral administration and its stability after infinite dilutions [42]. To study the robustness of formulations on dilutions, 100 mg of s-SNEDDS were diluted with deionized water to 50, 100 and 200 folds separately and gently shaken and sonicated for 50 s to prevent bubbles from interfering in the observation and left undisturbed for 2 h. Then, 1 mL of sample was removed and poured into the cuvette for measurement. Droplet size, PDI and percentage of transmittance was measured.

#### 2.8.5. Drugs Content Analysis

For the estimation of drug content in the formulation, 100 mg of s-SNEDDS were added in the mixture of ACN and methanol and vortexed for 15 min. Then, the vortexed mixture was sonicated for 20 min for the proper extraction of the drugs [43]. Further, the resultant extracted mixture was centrifuged for 10 min and then the collected supernatant was diluted and quantified for TAM and RES using the developed HPLC method. Before this study, the HPLC method was developed for drug content analysis using the C_8_ analytical column along with ACN:0.1% TFA (50:50) as the mobile phase with 0.7 mL/min of flow rate at the wavelength of 265 nm, where the retention time of TAM and RES was found to be 16.725 min and 5.725 min, respectively. All the observations were conducted in triplicate.

#### 2.8.6. Morphological Analysis

##### Scanning Electron Microscope (SEM)

The scanning electron microscope (SEM) (Zeiss, Germany) was used to determine the surface morphology of TAM, RES, the pure solid carrier neusilin US2 and s-SNEDDS. All samples were spread over the double-sided bi-adhesive carbon tape, which was attached with a metallic stud. Then, the platinum coating under an argon atmosphere using an ion sputter at 15 mA were applied over the samples and visualized under accelerating voltage ranging from 2–5 KV [44].

##### Transmission Electron Microscope (TEM)

The shape and size of droplets produced following the reconstitution of s-SNEDDS were determined using TEM. A single drop of reconstituted sample was placed on a 200 mesh carbon coated grid and then stained with 2% uranyl acetate. After drying, the grid with stained samples was visualized under TEM (FEI Company, USA) at an accelerating voltage of 100 KV [40].

#### 2.8.7. Solid State Characterization

##### Differential Scanning Calorimetry (DSC)

The DSC analysis was used to study the enthalpy change and physical state of TAM and RES in the optimized formulation. For this, 2 mg of TAM, RES, their mixture, neusilin US2 and s-SNEDDS were weighed accurately and sealed in aluminum hermetic pans. Then, the pans were analyzed by using Perkin Elmer Pyris 6 DSC (Waltham, MA, USA) at 10 °C/min of heating rate ranging from 50–450 °C in the presence of nitrogen gas with 25 mL/min of flow rate [44].

##### X-ray Diffraction (XRD)

The XRD pattern of TAM, RES, their mixture, neusilin US2 and s-SNEDDS were determined using Malvern Panalytical XRD equipment (Empyrean XRD Worcestershire, UK). The analysis of the samples was carried out in the presence of a graphite crystal monochromator with a filter radiation of Cu- Kα1 (γ = 1.5406 A°) at 30 KV with 30 mA and a diffraction angle (2 θ) was in the range from 5° to 50° with a scanning speed of 1.2°/min to accurately measure the crystallinity of the samples [45].

##### Fourier Transform Infrared (FTIR)

The Fourier Transform Infrared (FTIR) spectra of TAM, RES, their mixture, neusilin US2, and s- SNEDDS were recorded using a FTIR spectrometer (InfraRed Bruker tensor 37, Billerica, MA, USA). For this, 2 mg of each sample were mixed with 100 mg of pure potassium bromide separately and the mixtures were compressed into a disc with the help of a hydraulic pressure machine at a pressure of 5 t. The spectra with 32 scans were recorded within the range from 400–500 cm^−1^ wave number to check the interaction between drugs and excipients [44].

### 2.9. In Vitro Studies

#### 2.9.1. Reconstitution Ability and Stability of s-SNEDDS in Simulated Gastrointestinal Fluids

The reconstitution ability and stability of formulation in different digestive fluids were studied by adding 100 mg of s-SNEDDS to 90 mL of simulated gastric fluid (SGF at pH 1.2) and simulated intestinal fluid (SIF at pH 6.8), respectively. Then, mixtures were vortexed for 15 min and sonicated only for 50 s to prevent bubbles from interfering in the observation. For stability, the mixtures were kept in an incubator for 2 h in case of SGF, while for 6 h in case of SIF [46]. The obtained nanoemulsions in both cases were analyzed for droplet size, PDI and percentage of transmittance. All the observations were conducted in triplicate.

#### 2.9.2. Release Studies Using Dialysis Membrane

The in vitro release of drugs from TAM-RES-s-SNEDDS and Tam-RES-suspension was conducted using the dialysis bag method. TAM and RES-loaded s-SNEDDS and suspension were filled in a pre-activated dialysis bag (molecular weight cutoff 12 kDa) separately. Then, all individual dialysis bags were suspended in 25 mL of SGF of pH 1.2 and 25 mL of SIF of pH6.8 separately for 12 h. The whole study was conducted on a shaker water bath maintained at 37 ± 2 °C and 100 strokes/min with release media containing 10% Tween 80. The samples of 1 mL were collected at predetermined time points (0.5, 1, 2, 3, 4, 6, 8 and 9 h) and replaced with fresh media of the same volume [46,47]. Further, the content of drug release from all samples were quantified using the developed HPLC method. Fitting the data to kinetics models such as zero order, first order, Higuchi’s Korsmeyer–Peppas and Hixon–Crowell revealed the drug release mechanism and kinetics.

#### 2.9.3. Non-Everted Gut Sac Permeability Study

To assess the difference between permeation rate and absorption efficacy from optimized SNEDDS formulation and suspension, a non-everted gut sac study was performed using rat intestines. The isolated intestinal part was washed between 6 and 8 times with Tyrode’s solution. The separated segments were ligated from one end and filled separately with formulation, i.e., SNEDDS and suspension, and ligated from the other side to form a non-everted gut sac [24,48]. The non-everted sac filled with formulation and suspension were immersed in 25 mL of Tyrode’s solution maintained at 37 ± 2 °C with a continuous supply of oxygen. The solution present outside the sac is referred to as serosal fluid and the solution inside the sac is referred to as mucosal fluid [49]. The serosal fluid was collected at the predetermined time interval of 0.33, 0.66, 1, 1.33, 1.66 and 2 h and the content of the drugs was estimated by the in house HPLC method.

#### 2.9.4. Assessment of Depth of Permeation Using Confocal Laser Scanning Microscopy (CLSM)

The depth of internalization of SNEDDS loaded with drugs in the intestine was determined by the confocal laser scanning microscopy (CLSM) imaging tool. This study was also performed using rat intestines. TAM and RES-loaded SNEDDS and suspension were loaded with rhodamine-B (ROD-B) dye by mixing at the time of preparation. They were filled in the intestinal segment and both ends were tightly ligated to form a sac. Then, the sac of SNEDDS and suspension were placed in the pre-warmed Tyrode’s solution at 37 ± 2 °C, which was constantly stirred at a speed of 45 rpm with a continuous supply of oxygen. After between 4 and 5 h, the intestinal sac was removed and cut longitudinally and washed thoroughly with Tyrode’s solution to clean the surface from the free dye. Then, the small piece of the sac was cut and mounted on the slide to observe the extent of permeation of SNEDDS and suspension in the wall of the intestine by CLSM (Olympus Fluoview TM FV 1000) [29,50].

#### 2.9.5. In Vitro Lipolysis by pH Stat Method

To study the rate of lipolysis or impact of SNEDDS composition on the digestion of the lipid, a pH stat method was adopted as reported by Kumar et al. [51]. A digestion buffer of pH 6.8 was freshly prepared by adding CaCl_2_.2H_2_O (5 mM), NaCl (150 mM), tris maleate (150 mM) and NaOH (39.75 mM). For the lipolysis medium, to 100 mL of digestion buffer of pH 6.8, 0.26 g of taurocholic acid (5 mM) and 0.10 g of L-α-phosphatidylcholine (1.25 mM) were added and heated at 50 °C and stirred until all ingredients were fully dissolved. At the beginning of the experiment, to 35.5 mL of lipolysis medium, 1 g of SNEDDS was dispersed and stirred continuously with the maintained pH of 6.8 by using NaOH and HCl. Then, after 15 min, 3.5 mL of pancreatin extract was added, which started the enzymatic digestion of the lipids. At the time of the lipolysis process, the free fatty acids (FFA) were released, which subsequently reduced the pH of the medium. This was maintained at 6.8 by adding NaOH. The end of the enzymatic digestion process of the lipids was shown by the completion of pH titration. The final volume of NaOH utilized to maintain the pH at 6.8 throughout the experiment was recorded to calculate the released FFA during the lipolysis study by using Equation (4):

% FFA = 100 × (V_NaOH_ × M_NaOH_ × M_Lipid_)/W_lipid_ × 2 (4)

where V_NaOH_ is the volume of NaOH required to neutralize the released free fatty acids and M_NaOH_ is the molarity of NaOH_,_ M_Lipid_ is the molecular mass of the triacylglycerol oil and W_lipid_ is the total mass of triacylglycerol oil initially present for the study.

At the end of the lipolysis process, the medium was centrifuged at 1300 rpm for 30 min for the separation of the aqueous and sediment phase, which were quantified for drug content. The aqueous phase is comprised of monoglycerides, bile salts and fatty acids, whereas the sediment phase is comprised of undissolved fatty acids [52].

#### 2.9.6. Hemolysis Test

Hemolysis is known as the process of rupture and dissolution of red blood cells (RBCs). It is a common blood problem caused by nanoformulations loaded with chemotherapeutic drugs. Thus, a hemolysis test is usually used to evaluate the degree of RBCs destruction caused by nanoformulation and to estimate the amount of haemoglobin released after RBCs damage. The hemocompatibility of SNEDDS was determined by a hemolysis test [53]. Blood from a healthy Wistar rat was collected in an anti-coagulant tube and washed three times with normal saline and centrifuged at 3000 rpm for 10 min [54]. RBCs pellets were washed continuously until the clear supernatant was observed. Then, RBCs pellets were diluted with normal saline to form a suspension. To 180 µL of RBCs suspension, 20 µL of tested samples including SNEDDS (1:10), suspension (1:10), placebo, phosphate buffer saline (PBS) and triton X 100 were added, respectively. For morphological assessment, the mixtures were incubated for 2 h and visualized under microscope.

For the percentage of hemolysis, the mixture was stabilized at 37 ± 2 °C for 2 h in a water bath and centrifuged at 3000 rpm for 15 min. The collected supernatant was measured for absorbance at 404 nm by a microplate reader. PBS and triton X 100 were considered as negative and positive controls [55].

### 2.10. Cell Line Studies

#### 2.10.1. Cytotoxicity Studies

The cell plates were prepared as per the method described in Section 2.2 and allowed to attain 90% confluency of cells. s-SNEDDS and suspension were diluted with fresh media to attain the concentration of 0.65. 1.25, 2.5, 5 µg/mL and incubated with the MCF-7 cells for 12 h.

The each well of the pre-treated plate were again treated with 20 µL of MTT solution (5 mg/mL in PBS pH 7.4) and incubated for 4 h at 37 ± 2 °C for the formazan crystals formation that were dissolved in 150 µL of DMSO on mechanical plate mixer. Then, the optical density was measured at 570 nm on an ELISA reader plate (Synergy HT, BioTek, Winooski, VT, USA). The percentage of cytotoxicity of all the formulations were evaluated.

#### 2.10.2. Cellular Uptake Studies

The cellular uptake of formulation was studied on the MCF-7 cell line using fluorescence microscopy. TAM and RES-loaded SNEDDS and suspension were prepared and labeled with ROD-B dye by mixing at the time of preparation. For this study, a circular glass coverslip 12 mm in size was placed in the 24-well microplate and seeded with MCF-7 cells with a density of 1 × 10^4^ cells/well. Each well was supplemented with 10% fetal bovine serum (FBS), 100 units/mL of penicillin and 100 mg/mL of streptomycin followed by incubation of 24 h at 37 ± 2 °C with an atmosphere of 5% CO_2_. Then, after the attainment of 90% confluency, the cells were treated with labeled SNEDDS and suspension and incubated for 4 h. After the incubation, the cells were twice washed with ice cold phosphate buffer saline to remove uninternalized nanoparticles. Then, the cells were fixed with 4% formaldehyde and mounted on a glass slide by using a DPX mounting medium. The slides were visualized by using a Fluorescent upright microscope (Eclipse 90i, Nikon Corporation, Tokyo, Japan) with a red filter, which is specific for ROD-B dye [56].

#### 2.10.3. Estimation of Intracellular Antioxidant

##### Reactive Oxygen Species (ROS) in MCF-7 Cells by DCFDA Assay

The redox state is disturbed either by the over production of ROS or reduction in the antioxidant mechanism leading to an oxidative stress state which leads to DNA damage, cell mutation, cell inflammation and cell proliferation, which further results into carcinogenesis [57]. Moreover, the ROS is the major risk factor for the development and progression of breast cancer. To determine the antioxidant potential of the formulation, levels of ROS were estimated by using the 2,7-Dichlorofloresin diacetate (DCFDA) method [58]. This was performed in a 96-well culture plate seeded with 2 × 10^5^ MCF 7 cells/well. Then, cells were treated with SNEDDS and suspension and incubated for 24 h. After incubation, the cells were treated with the DCFDA florescent dye (25 μM) for 30 min at 37 ± 2 °C in the dark for the evaluation of intracellular ROS generation. Further, the wells were rinsed with chilled PBS (Phosphate Buffer Saline, 0.02 M, pH 7.4). Then, the intensity of fluorescence was quantified by using a spectrofluorimeter (Carl Zeiss, Oberkochen, Germany) with excitation and emission wavelengths of 488 nm and 522 nm, respectively.

##### Assessment of Superoxide Dismutase Activity (SOD)

Antioxidants are of two types including endogenous (inside body) and exogenous (outside body). Three primary endogenous antioxidant enzymes are superoxide dismutase (SOD), catalase and substrate specific peroxide. The SOD converts ROS into hydrogen peroxides and molecular oxygen, whereas catalase and peroxides convert hydrogen peroxides into water. To estimate the SOD activity after treatment, the study was conducted on cell-free extracts (CFE). Briefly, MCF-7 cells in the density of 1 × 10^6^ cells were seeded in a 6-well plate in DMEM, supplemented with 10% fetal bovine serum (FBS) and incubated for 24 h at 37 °C with an atmosphere of 5% CO_2_. After incubation, the cells were treated with TAM and RES-loaded SNEDDS and suspension for 24 h. After sufficient treatment, the pellets were collected via centrifugation and resuspended into 200 µL of chilled PBS. The suspension of cells was sonicated for 3 min, followed by syringe pipetting, so as to break the cells and release the cytoplasmic contents. The cell-free extract (CFE) was collected after centrifugation and used for estimating the level of SOD activity. The temperature of 10 ± 2 °C was maintained throughout the experiment.

Kepinska protocol for the measurement of SOD activity was followed in this study [59]. Used in the synthesis were 0.025 M sodium pyrophosphate (pH 8), 180 µM phenazine methosulfate and 300 µM nitroblue tetrazolium (NBT). In the final assay mixture of 100 µL, 20 µL of CFE was added together with 60 µL of reaction mixture, and the reaction was started by adding 20 µL of 780 µM NADH. The absorbance was measured at 560 nm for 5 min immediately against a blank containing phosphate buffer instead of the sample. SOD activity was measured in units/mg of protein [60].

### 2.11. Pharmacokinetic Study

Albino Wistar rats of either sex were issued and used for the study after taking prior approval from the Institutional Animal Ethical Committee (IAEC), Jamia Hamdard, New Delhi, with protocol number 1471. Animals were housed in polypropylene cages under standard laboratory conditions at a temperature of 25 ± 2 °C with a relative humidity between 50 and 60% with free access to water.

The rats were randomly divided into three groups. The rats of group 1 received a TAM suspension at a dose of 1.02 mg/kg, group 2 received a TAM (1.02 mg/kg) and RES- (10.20 mg/kg) loaded suspension and group 3 received a TAM (1.02 mg/kg) and RES- (10.20 mg/kg) loaded SNEDDS. TAM and RES are insoluble in water, so their suspension was developed by mixing them with 0.25% of CMC-Na with constant stirring for 15 min. Blood samples were collected at predetermined time intervals of 0.5, 1, 1.5, 3, 5, 8, 10 and 24 h from all groups. The collected samples were centrifuged at 5000 rpm at 4 °C for 15 min for the separation of plasma. Then, 50 µL of plasma samples were mixed with 50 µL of internal standard (IS) (1-amino-4-nitro naphthalene of 100 µg/mL) and vortexed for 10 min. Subsequently, equal amounts of ACN was added and vortexed for 15 min to precipitate the plasma protein. The mixtures were centrifuged at 5000 rpm for 15 min to collect the supernatant. The extracted drug was quantified by the developed and validated UPLC- MS/MS method. The pharmacokinetic parameters for the TAM and RES-loaded suspension and SNEDSS were estimated.

UPLC-MS/MS analysis was performed by using an instrument, Waters Acquity UPLC-MS/MS system (Waters, XEVO-TQD, Milford, MA, USA), including the binary solvent manager. The instrument was equipped with a triple quadrupole mass spectrometer operated in multiple reaction monitoring (MRM) mode for selective quantification of TAM and RES in plasma samples. All data acquisition and peak integration in MRM were performed using Masslynx software (versions 4.1) from Waters (Milford, MA, USA). Optimization of MS parameters was conducted before analysis including ionization mode, transition of parent to daughter ions, Collision Energy (CE), Cone Voltage (CV) and dwell time. Ionization of TAM and IS was optimized in ESI positive mode, whereas RES was better ionized in negative ionization mode. The source temperature was maintained at 130 °C, dissolvation temperature was 350 °C, dissolvation gas was nitrogen at 850 L/h, cone gas was at 50 L/h and capillary voltage was 3.5 KV for both the modes. The CE for TAM, RES and IS were optimized as 25 V, 15 V and 15 V, respectively. Similarly, CV was optimized for TAM, RES and IS as 40 V. The ion transitions were selected for the quantification of TAM was 372.4 > 71.9; RES was 227 > 185.1; IS was 189.1 > 172.1. All the chromatographic separations were performed on Acquity UPLC BEH C_18_ column (2.1 mm × 100 mm) with 1.7 µm particle size and a maintained temperature of 40 °C. The mobile phase consisted of a mixture of acetonitrile and 2 mM of ammonium acetate in the ratio of 80:20 at the flow rate of 0.4 mL/min. The total run time was 3 min.

### 2.12. Statistical Analysis

All the experiments were performed in triplicates, and all data are presented as mean ± SD. Statistical analysis was performed using one-way analysis of variance with the level of statistical significance set at *p* < 0.05.

## 3. Results and Discussions

### 3.1. Determination of Combination Index (CI)

**Figure 2:**
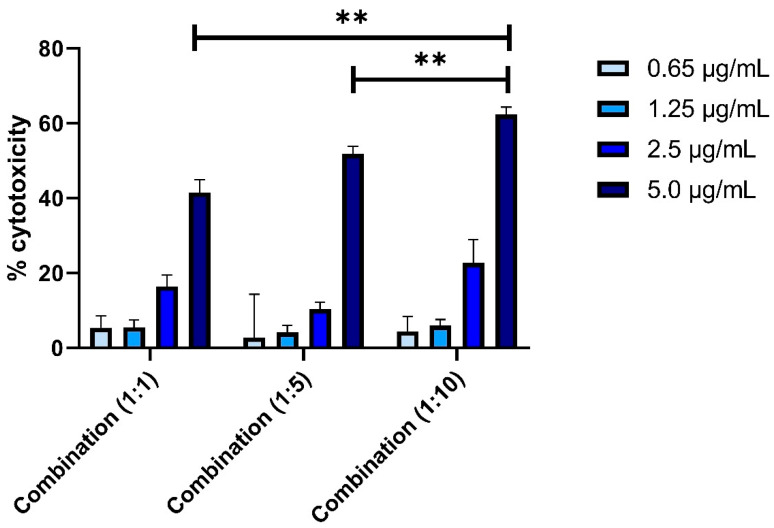
Cytotoxicity of TAM and RES combination in different ratios (1:1; 1:5; 1:10). Data are given as mean ± standard deviation (*n* = 3) with statistical difference at (**) *p* < 0.01.

After observing the effect of isolated drugs and evaluating their IC_50_ values, the drugs were studied in association with each other in different ratios. The studies were carried out on MCF-7 with varied concentration (0.65, 1.25, 2.5 and 5 µg/mL) of TAM and RES combinations in different ratios, namely 1:1, 1:5 and 1:10. There was an increase in the cytotoxicity in all the combinations in a dose-dependent manner (Figure 2). The IC_50_ of TAM was found to be 7.422 µg/mL, which was quite high in comparison to the IC50 of TAM in combination with RES in different ratios (1:1), (1:5) and (1: 10), which were 2.946, 0.853 and 0.391 µg/mL, respectively. The results indicated that the IC50 of TAM was continuously decreased with an increase in the RES part in the combination. Hence, the cytotoxicity of TAM was improved with RES in combination. TAM is an estrogen receptor blocker and can act as an anticancer agent, whereas the RES is a natural antioxidant, having anticancer action by initiation, progression and suppression of carcinogenesis, which ultimately synergizes the anticancer action of other drugs. For the type of association between the two drugs, the CI for (1:1), (1:5) and (1: 10) were found to be 0.444, 0.182 and 0.116, respectively. The results showed significantly (*p* < 0.01) high cytotoxicity with a 1:10 ratio due to a high amount of RES along with TAM, which showed highly synergized anticancer action in MCF-7 (Figure 2). Thus, due to maximum toxicity, the combination with a drugs ratio of 1:10 were selected for formulation development and further characterization studies.

### 3.2. Selection of Excipients

#### 3.2.1. Selection of Oil

**Figure 3:**
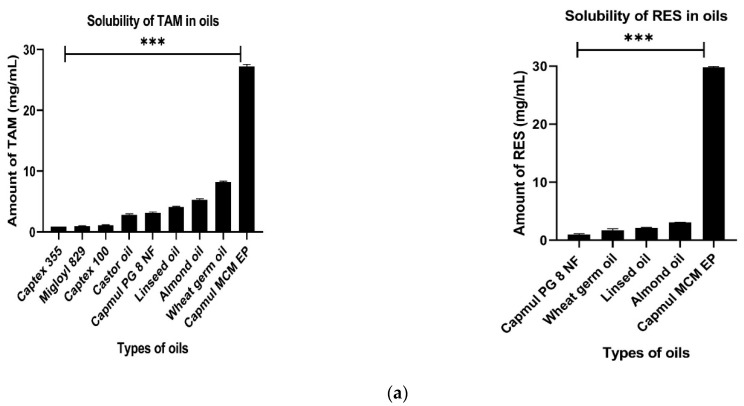

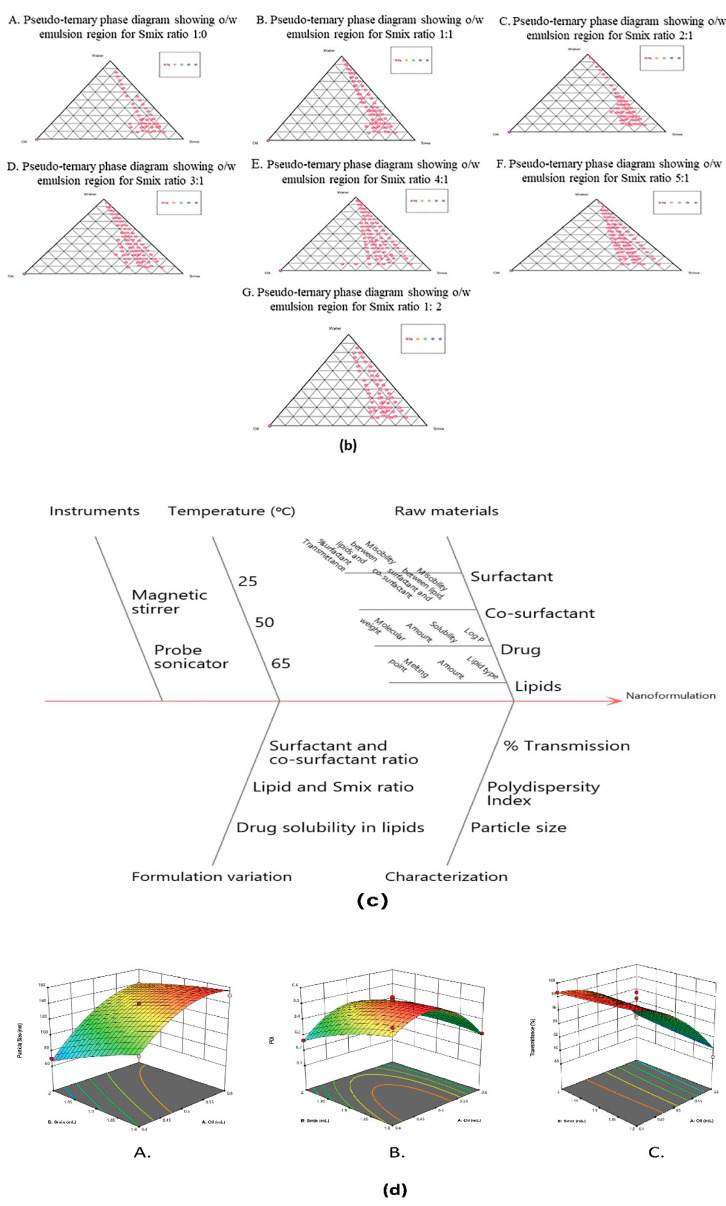
Representation of (**a**) solubility of TAM and RES in various lipids. Maximum solubility of both drugs was observed in capmul MCM EP. (**b**) Pseudo ternary phase diagram with different Smix ratios 1:0, 1:1, 2:1, 3:1, 4:1, 5:1, 1:2. (**c**) Ishikawa diagram for SNEDDS development. (**d**) 3D surface response plot showing effect of concentration of oil and S_mix_ on the (**A**). Droplet size—Droplet size increased with an increase in the concentration of oil whereas, decreased with an increase in the concentration of S_mix_, (**B**). PDI—PDI decreased with an increase in the concentration of oil and increased with an increase in the concentration of S_mix_. However, combination of concentration of oil and S_mix_ provided better homogeneity (**C**). % Transmittance—% Transmittance increased with decrease in the concentration of oil and increase in concentration of S_mix_. Data are given as mean ± standard deviation (*n* = 3) with statistical difference at (***) *p* < 0.001.

TAM and RES showed maximum solubility in capmul MCM EP, i.e., 27.318 ± 0.31 mg/mL and 29.73 ± 0.58 mg/mL, respectively (Figure 3a). Capmul MCM EP is a semisynthetic medium chain triglyceride and can be easily emulsified in an aqueous medium due to lower interfacial tension, enhanced water solubility and better partitioning ability [61]. Major surfactants are not able to emulsify the oil with higher molecular weight due to a longer hydrophobic alkyl chain in comparison to medium chain triglycerides (Capmul MCM EP) in which the surfactant can easily incorporate their long-chain fatty acids within the core of the oil globules [38].

#### 3.2.2. Selection of Surfactant

The selection of the surfactant and co-surfactant was conducted on the basis of its emulsification ability with the selected oil, i.e., capmul MCM EP. The four surfactants screened were labrafac lipophile WL 1349, caproyl PGMS, cremophore and Tween 80. Amongst all, Tween 80 was selected, as it resulted in the formation of a clear emulsion with oil and had 75 ± 0.2% T. Tween 80 is a promising surfactant due to its high HLB value, i.e., 15 in comparison to the HLB value of capmul MCM EP, i.e., 5.5 due to which it exhibits a better emulsifying property that resulted in a decrease in interfacial tension, low entropy and rapid dispersion of oil globules in the aqueous phase that ultimately provided a stable microscopic oil in water nanoemulsion [62]. Since Tween 80 is non-ionic in nature, it results in a low magnitude of charge over the nanoparticles, which reduces the chances of agglomeration leading to stabilization of the formulation [63]. It is reported as less toxic and the preferred surfactant for oral ingestion [64].

Co-surfactants help to stabilize the nanoformulations by lowering the interfacial tension and by changing the curvature of the reverse micelles. Transcutol HP was selected as a co-surfactant as it emulsified both the selected oil and the surfactant and resulted in a transparent emulsion with transmittance greater than 90 ± 0.3%. It is a promising co-surfactant as it helps to stabilize the interfacial film along with the surfactant due to its low HLB value of 4.2 [65].

#### 3.2.3. Pseudoternary Phase Diagram

To select the optimum ratio of surfactant:co-surfactant and oil:S_mix_, a pseudoternary phase diagram consisting of capmul MCM EP, S_mix_ (Tween 80 and transcutol HP) along with deionized water was constructed (Figure 3b). These diagrams were made on the basis of the largest isotropic nanoemulsion region, and it helped in giving an indication about the self-nanoemulsifying efficiency of S_mix_, which is directly proportional to the size of the nanoemulsion region. The self-nanoemulsifying region is an area, where on dilution, clear and transparent formulations are obtained. When a 1:0 ratio of S_mix_ was titrated, the emulsion obtained was not a clear emulsion, which indicated that Tween 80 alone was not able to completely emulsify capmul MCM EP. In the case of a S_mix_ ratio of 1:1, a small emulsion region was obtained after dilution with deionized water. It indicated that the S_mix_ of 1:1 was not able to completely emulsify the oil phase. As the S_mix_ ratio increased from 2:1 to 3:1, the nanoemulsion region also increased. In a S_mix ratio_ of 4:1, the maximum nanoemulsion region was obtained due to an increase in self-emulsification, decreased interfacial tension and rapid dispersion of oil in the aqueous phase. However, with the increment in the S_mix_ to a ratio of 5:1, the nanoemulsion region reduced, which indicated that a further increase in the surfactant concentration did not enhance the emulsification region [66]. In the case of the S_mix_ ratio of 1:2, the decreased nanoemulsion region obtained with slimy liquid phase indicated that more surfactant was necessary to emulsify the oil phase. Furthermore, the S_mix_ of 4:1 showed significant increase in the nanoemulsion region with oil until 1:4 (oil: S_mix_). However, further addition of the oil, i.e., more than 50% of the total composition of formulation, resulted in the turbidity of nanoemulsion. One more important observation was that the nanoemulsion formed with selected ratios of excipients was stable and transparent after storage for 24 h.

### 3.3. QbD Approach

The CQAs of the present study were defined as droplet size, polydispersity index and percentage of transmittance as they could have affected the final quality of developed SNEDDS. The risk assessment was carried out by drawing an Ishikawa diagram (Figure 3c), which helped to understand the effect of each variable on CQAs.

### 3.4. Optimization of SNEDDS Using CCRD

The Design Expert^®^ software suggested 13 runs with variable concentrations of oil and S_mix_. As per the suggested runs, different formulations were obtained and evaluated for the effect of independent variables, such as concentration of oil and S_mix_, on the dependent variables of droplet size, PDI and percentage of transmittance. All the observed responses obtained from the 13 runs were recorded and used for ANOVA application to determine the significant model.

#### 3.4.1. Effect of Independent Variables on Droplet Size

The droplet size is the key parameter for the nanoemulsion because it is related to the release of drugs. The smaller the droplet size, the larger the surface area will be for more drug release and ultimately the enhanced oral bioavailability of the drugs from the nanoemulsion to the body for its therapeutic action [67]. The droplet size in all experimental runs was within the range from 60.18–160.20 nm. The results were best-fitted in a quadratic model with an F value of 84.90, which implied the model was significant. The R^2^ value was found to be 0.9838, indicating a strong relationship between the process variables and the observed results. The strong influencing factors for droplet size were the concentration of oil and S_mix_, as represented by Equation (5). Droplet size increased with an increase in the concentration of oil and decreased with an increase in the concentration of S_mix_, as shown in {Figure 3dA). The results depicted a 5% level of significance and revealed a direct relationship with the concentration of oil and inverse relationship with the concentration of S_mix_. A high concentration of surfactant stabilizes the formulation and creates a barrier at the surface of nanoemulsion and prevents their agglomeration into larger droplet sizes [29]. It was observed that using a lower percentage of oil in the formulation, i.e., less than 4%, resulted in smaller droplet sizes.

Droplet size (Y1) = + 138 + 29.66 × X1 − 15.09 × X2 + 7.50 × X1X2 − 17.13 × X1^2^ − 2.12 × X2^2^ (5)

#### 3.4.2. Effect of Independent Variables on Polydispersity (PDI)

PDI provides an indication about the homogeneity of particle distribution in the formulation. The average dispersity in all experimental runs ranged from 0.195–0.543, indicating a homogenous distribution profile. The results were best-fitted in a quadratic model with an F value of 15.71, which implied that the model was significant. The influence of the concentration of oil and surfactant on polydispersity is represented by polynomial Equation (6) and was found to have a level of significance less than 5%.

PDI (Y2) = 0.5134 − 0.0270 × X1 − 0.0483 × X2 + 0.0483 × X1X2 − 0.1536 × X1^2^ − 0.0273 × X2^2^ (6)

The R^2^ value was found to be 0.9182, depicting a good correlation between the process variables and obtained response. The analysis of polydispersity confirmed a negative relationship with the concentration of oil and a positive relationship with the concentration of S_mix_ (Figure 3dB). In addition, the combination of concentration of oil and S_mix_ provided better homogeneity, which resulted in uniformly distributed spherical particles. Mainly, the optimum ratio of surfactant and co-surfactant, i.e., S_mix_, is responsible for uniformity in the nanoemulsion particles [68]. The increment in surfactant concentration leads to the decrease in polydispersity because of complete coverage around the drug, whereas a slight decrease also increases the droplet size. Uniformity of the particles is the significant quality of the formulation as these particles accumulate at the site of absorption and results in expected pharmacological action [69].

#### 3.4.3. Effect of Independent Variables on Percentage of Transmittance

In all experimental runs, the percentage of transmittance ranged from 88.79–99.25%, indicating the transparency of the nanoformulation. The results were best-fitted in a quadratic model with an F value of 13.95, which implied that the model was significant. The influence of the concentration of oil and surfactant on the percentage of transmittance was described by polynomial Equation (7):

% transmittance (Y3) = + 96.72 − 3.97 × X1+ 0.1355 × X2 + 0.5773 × X1X2 − 1.69 × X1^2^ + 0.069 × X2 (7)

The R^2^ value was found to be 0.9088, depicting a good correlation between the process variables and obtained response. The analysis of the percentage of transmittance confirmed the negative relationship with the concentration of oil and the positive relationship with the concentration of S_mix_ (Figure 3dC). The higher value of percentage of transmittance indicated transparency, having homogenous size distribution, whereas the lower value indicated the turbid formulation.

#### 3.4.4. Validation of Experimental Design

The CCRD predicted optimum formulation responses, which were then correlated with the obtained results. The optimized formulation consisted of 0.600 mL of oil and 1.860 mL of Smix. The predicted response of droplet size, PDI and % transmittance was 111 nm, 0.256 and 95%, respectively. While the response obtained after using this composition was found to be 104.5 nm, 0.211 and 94%, respectively. The results suggested that a good correlation existed between the predicted and obtained responses, which established the accuracy in the formulation of SNEDDS. The responses of the optimized formulation were generated with a desirability factor close to 1.

### 3.5. Conversion of SNEDDS into Solid SNEDDS

The optimized amount of neusilin US2 was found to be 600 mg for the adsorption of optimized liquid SNEDDS. The s-SNEDDs developed was free flowing and reconstituted within 5 min.

### 3.6. Characterization of s-SNEDDS

#### 3.6.1. Droplet Size and PDI

**Figure 4:**
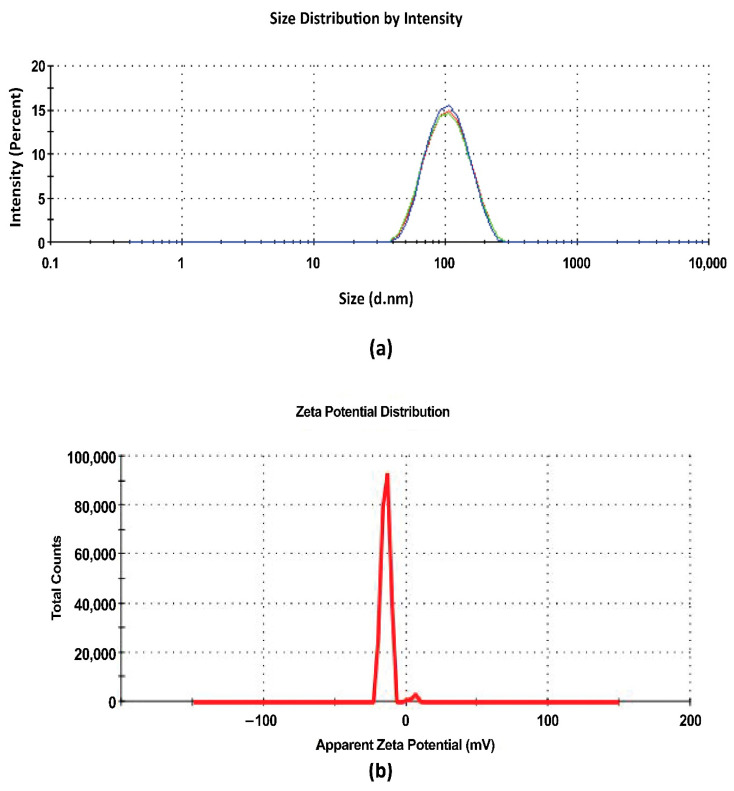
Graphical representation of (**a**) droplet size and (**b**) zeta potential of TAM-RES-s-SNEDDS.

The droplet size of TAM-RES-s-SNEDDS were found to be 92.54 ± 3.98 nm (Figure 4a). The smaller droplet size enhances the penetration of drugs via the epithelial layer resulting in enhanced permeation across the intestinal membrane and ultimately increasing the bioavailability of the drugs loaded in SNEDDS. The nanoemulsified drugs can efficiently enter into the cytoplasm and into more specific sites so as to provide more cytotoxicity in the case of cancer cells. Further, the droplet size of nanocarriers must fall between the range 50 nm and 200 nm because the nanocarriers with a size below 50 nm are mostly eliminated by the kidney, whereas the nanocarriers with a size above 200 nm undergo mononuclear phagocyte system (MPS) uptake [70]. The droplet size of optimized SNEDDS was more than 50 and less than 100 nm, which indicated the enhanced and efficient deposition of nanocarriers to the target site, i.e., breast cancer cells [71]. It was observed that small droplet size was due to the high concentration of S_mix_ at the interface of oil and water, which provided a strong mechanical barrier against the aggregation of the globules along with a reduction in the free energy of the system, hence, promoting the stability of the system in the changing environment [72].

PDI measures the uniform homogeneity of the globules. The lower the PDI, the more uniform the homogeneous distribution of oil globules in the aqueous phase. The PDI of TAM-RES-s-SNEDDS were found to be 0.208 ± 0.012. The lower PDI of the SNEDDS was due to the optimum ratio of surfactant and co-surfactant. Due to this optimized ratio, the interfacial tension was reduced to the maximum extent, which prevented the aggregation of oil globules and resulted in the thermodynamically stable formulation with uniform droplet size [72,73].

#### 3.6.2. Percentage Transmittance

The percentage of transmittance of TAM-RES-s-SNEDDS was found to be 96.00 ± 0.90. This highlights the significance of droplet size, which was in the nanometric range that provided a large surface area for drug release, thus, improving the bioavailability [65]. Additionally, these results indicated that the solid carrier, i.e., neusilin US2, did not affect the percentage of transmittance and isotropic nature of the SNEDDS, thus, signifying that the solid carrier only acted as an adsorbent and did not interact with formulation excipients.

#### 3.6.3. Zeta Potential

Zeta potential is the potential between the surface of the droplets and dispersion medium. It is an indicator of the stability of the formulation; a value more positive than +30 mV and more negative than −30 mV represent the stable formulation against the coalescence and separation of the formulation. Its value is estimated by measuring the electrophoretic mobility of droplets [74]. The zeta potential of TAM-RES-s-SNEDDs was found to be −13.5 ± 0.87 mV (Figure 4b), and no coagulation, flocculation and agglomeration occurred in the formulation [75].

#### 3.6.4. Robustness to Dilutions

This experiment was used to study the robustness of the formulation towards the 50-, 100- and 200-fold dilution. The results obtained after the dilution of s-SNEDDS to 50, 100, and 200 folds showed acceptable droplet size (less than 200 nm), PDI and %Transmittance, which indicates the uniform drug release in in vivo conditions (Table 2).

#### 3.6.5. Drugs Content Analysis

The content of TAM and RES estimated in s-SNEDDS was found to be 85.15 ± 0.58% and 89.47 ± 0.23%, respectively. The results indicated that a high concentration of S_mix_ and optimum ratio of oil and S_mix_ afforded the capacity to solubilize 10 mg of TAM along with 100 mg of resveratrol.

#### 3.6.6. Morphological Analysis

##### Scanning Electron Microscope (SEM)

**Figure 5:**
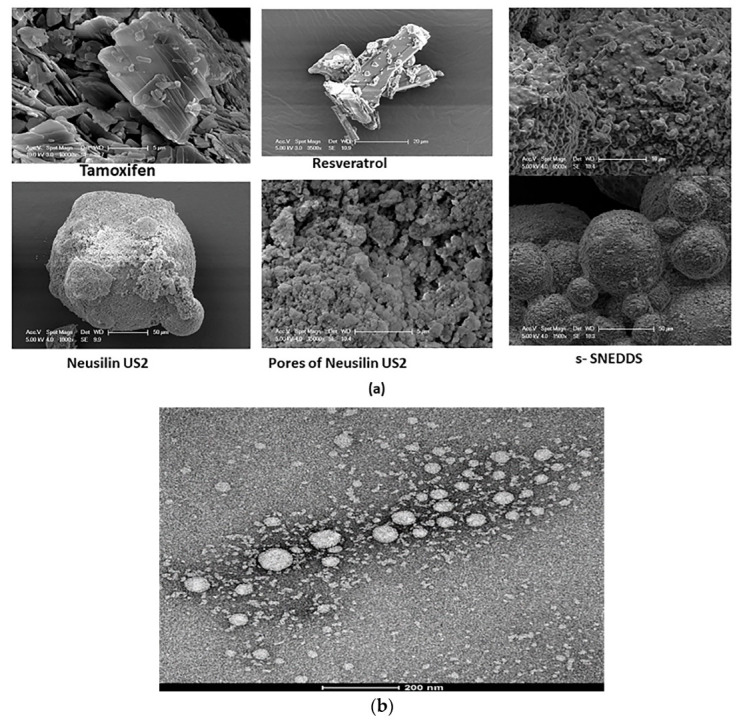
Surface morphology of (**a**) TAM, RES, neusilin US2 and s-SNEDDS by SEM. (**b**) s-SNEDDS by TEM depicting the spherical shape of the droplets.

The surface morphology of TAM, resveratrol, neusilin US2 and s-SNEDDS were studied using SEM (Figure 5a). The pure TAM was in crystal form with defined facets and sharp edges [76], whereas the pure RES was lacking uniformity in size and appeared large enough [77]. The neusilin US2 had a rough and porous spherical surface that was perfect for the ingress of lipidic formulation into the matrix. The s-SNEDDS showed spherically-shaped particles with a shallow cavity on the surface and appeared as an agglomerated mass. None of the drug crystals were observed on the surface of the solid SNEDDS, showing complete encapsulation of both drugs within the oil.

##### Transmission Electron Microscope (TEM)

Droplets obtained after the reconstitution of s-SNEDDS with deionized water showed a spherical shape with a droplet size of approximately 100 nm and verified the result obtained by the zeta sizer (Figure 5b). The droplets of developed nanoemulsion emerged as bright and surroundings were found to be dark indicating the formation of a thick monolayer around the emulsion droplets, which reduced the interfacial energy and resisted [48].

#### 3.6.7. Solid State Characterization

##### Differential Scanning Calorimetry (DSC)

**Figure 6:**
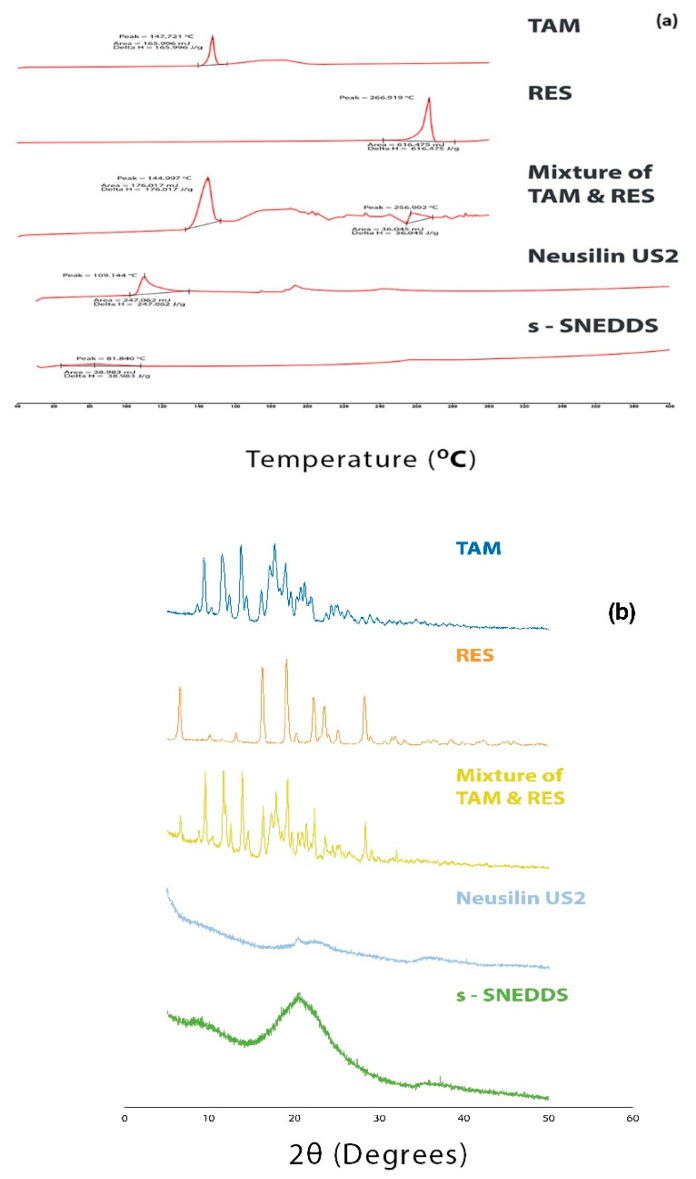

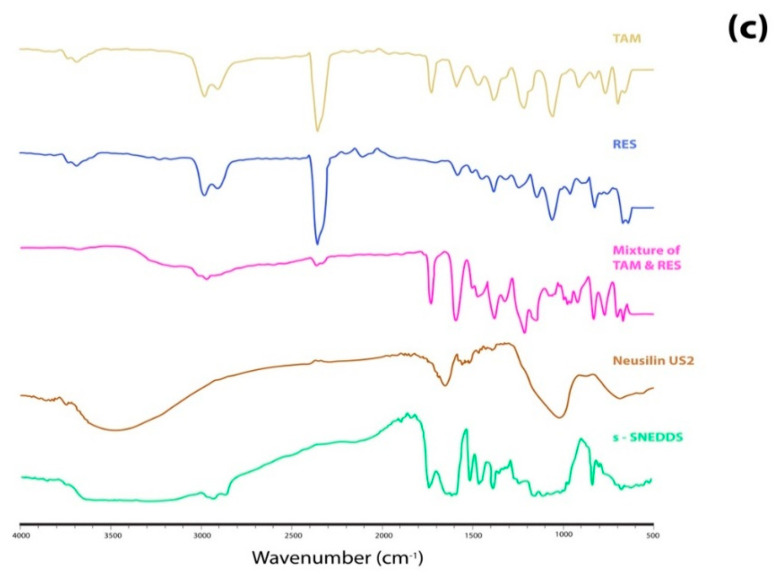
(**a**) DSC curve, (**b**) XRD graph, (**c**) FTIR spectra of TAM, RES Mixture of TAM and RES, Neusilin US2 and s-SNEDDS.

The DSC curve of TAM and RES (Figure 6a) showed a sharp endothermic peak (the point where the substance absorbs the heat, reaching its melting point, and changes from a crystalline to amorphous form) at 147.721 °C and 266.919 °C, which is same as reported in the literature [78,79]. The DSC curve of the mixture of both drugs (Figure 6a) also showed the same endothermic peaks with a slight shift in the height, shape and positions of the peaks due to the difference in the geometry of the mixture [80]. In the mixture, the drugs were present in their crystalline nature.

The DSC curve of s-SNEDDS (Figure 6a) showed a broad peak in comparison to the peak of neusilin US2 (Figure 6a) due to the loss of moisture content [81]. Moreover, the disappearance of characteristic peaks of both drugs was due to their complete transformation from a crystalline to amorphous form. Hence, it was concluded that both drugs were not found in crystalline form, which signifies that they were homogenously dispersed in the oil phase during the preparation of SNEDDS.

##### X-ray Diffraction (XRD)

The DSC results of the samples were further confirmed by the XRD analysis. The X-ray diffractograms of powder TAM, RES and their mixture are shown in Figure 6b. A sharp characteristic peak of TAM and RES was observed, indicating the presence of crystalline TAM and RES. The diffractogram of a mixture of TAM and RES showed the presence of characteristic peaks of both drugs with no new additional peaks, indicating that both drugs were present in their crystalline form in the mixture. A significant decrease in the degree of crystallinity was observed in the diffractogram of s-SNEDDS. These observations indicated that the drugs in s-SNEDDS were completely transformed into an amorphous form after being molecularly dispersed in oil and adsorbed over the porous surface of neusilin US2. The broad peak of s-SNEDDS may be due to the adsorption of liquid SNEDDS over the porous surface of neusilin US2.

##### Fourier Transform Infrared (FTIR)

The FTIR spectra of TAM, RES, their mixture, nesulin US2 and s-SNEDDS are shown in Figure 6c. Tamoxifen has a characteristic peak of -C=C- stretching, CH stretching and –NH2 at the frequency of 1728.27–1469.24 cm^−1^, 2976.35 cm^−1^ and 2976.35–3682.86 cm^−1^, respectively, whereas resveratrol has characteristic peaks of the -OH group, -C=O stretching, C=C at 2978.67 cm^−1^, 1583.16–2106.62 cm^−1^ and 1583.16–2106.62 cm^−1^, respectively. The combination of tamoxifen with resveratrol showed the presence of characteristic peaks of both drugs along with the broad peak of OH stretch at 2967.51 cm^−1^, which indicated that both drugs were not interacting. The characteristics peak of both drugs were retained in the formulation without any additional peaks confirming there was no interaction between the drugs and excipient [20].

### 3.7. In Vitro Studies

#### 3.7.1. Reconstitution Ability and Stability of s-SNEDDS in Simulated Gastrointestinal Fluids

The reconstitution ability and stability of s-SNEDDS in digestive fluids with or without an incubation period showed a reconstitution time of less than 5 min with minimum droplet size (<200 nm), narrow PDI (<0.2), and higher transmittance (>90%) (Table 3). Furthermore, no sign of drug precipitation or phase separation and turbidity was observed in both pH 1.2 and 6.8 with or without an incubation period. The results obtained indicated that the digestive fluids present inside the body did not affect the reconstitution behavior and stability of SNEDDS, which is very important as it affects the absorption and drug bioavailability in the body. In both cases, the emulsion developed with a small droplet size provided a large surface area for maximum drug release.

#### 3.7.2. Release Studies Using Dialysis Membrane

**Figure 7:**
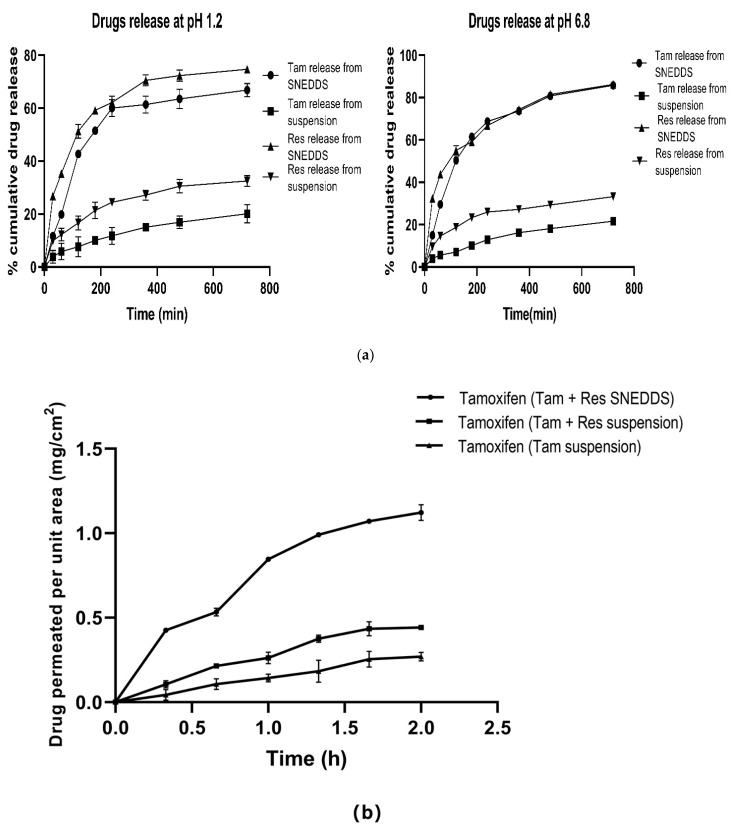

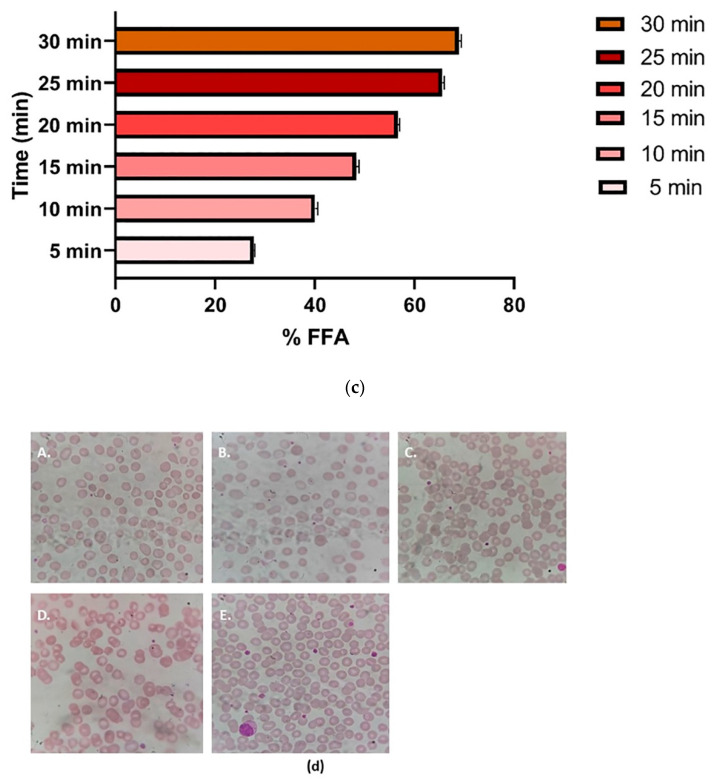
The in vitro (**a**) drug release profile of TAM and RES from s-SNEDDS and suspension in SGF (pH 1.2) and SIF (pH 6.8). (**b**) Permeation of TAM/area per unit time across non-everted gut sac from TAM-RES-s-SNEDDS, suspension and TAM suspension. (**c**) Lipolysis study showed % FFA release per unit time. (**d**) Morphology of RBCs after treatment with A. Suspension, B. Triton X100 as negative control, C. PBS as positive control, D. Placebo, E. SNEDDS.

The in vitro release of drugs from TAM-RES-s-SNEDDS and TAM-RES-suspension were performed in 25 mL of simulated gastric intestinal fluid with pH 1.2 and pH 6.8 separately. Within 60 min of the study, the percentage of drug release from the SNEDDS was observed to be 20%, which further reached 85% after 720 min and was found to be significantly higher (*p* ˂ 0.05) than the release of the drugs from the suspension (Figure 7a). This enhancement in the release of the drugs from the SNEDDS was due to the immediate formation of nanoemulsion with the nanometric droplet size range. Increased availability of both the drugs in the dissolved state could lead to the better absorption and enhanced bioavailability. Thus, the results of the in vitro drug release study indicated the enhanced solubility of TAM and RES in the selected oil and surfactant, i.e., capmul MCM and Tween 80 in the developed formulation [26]. The release kinetics of the optimized SNEDDS in SGF and SIF obeyed Higuchi’s diffusion model because its R^2^ value was close to 1. The other researchers also reported the similar kinetic model for the release of Olmesartan Medoxomil from SNEDDS [42].

#### 3.7.3. Non-Everted Gut Sac Permeability Study

The experiment was conducted to determine the permeation of TAM from dual drug-loaded SNEDDS and suspension, and TAM suspension across the membrane of the intestine. TAM and RES-loaded suspension and TAM suspension showed an apparent permeability coefficient (Papp) of 11.49 × 10^−5^ cm/s and 7.01 × 10^−5^ cm/s with a flux of 0.2298 mg/cm^2^/h and 0.1402 mg/cm^2^/h, respectively, whereas TAM-RES-s-SNEDDS showed a (Papp) of 27.4 × 10^−5^ cm/s with a flux of 0.5479 mg/cm^2^/h at 2 h. The permeability of TAM from the dual drug-loaded SNEDDS across the non-everted gut sac was found to be 2.3 times higher than that from the TAM and RES suspension, whereas the permeability of TAM from the combination suspension was observed to be 1.6 times higher than the TAM suspension, as shown in Figure 7b. The permeability of TAM from SNEDDS was found to be higher due to the nanometric droplet size with increased drug solubility, thus, enabling the drugs to easily pass through the intestinal wall from the mucosal to serosal side. Moreover, TAM is a P-gp substrate while RES is a potent P-gp inhibitor, therefore, the loading of RES in the formulation and suspension caused more release of TAM in comparison to the suspension of TAM alone. The previous literature also reported that the RES exhibited an inhibitory effect on the P-gp efflux pump and resulted in the enhanced permeation of fexofenadine [77,82]

#### 3.7.4. Assessment of Depth of Permeation Using Confocal Laser Scanning Microscopy (CLSM)

**Figure 8:**
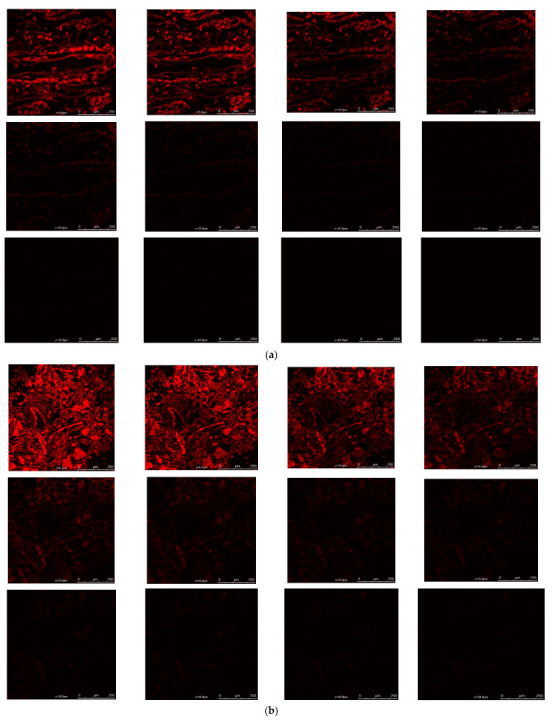
Confocal scanning images of drug permeation via (**a**) suspension and (**b**) SNEDDS across the intestinal membrane.

The permeation of SNEDDS and suspension via enterocytes of the intestinal lumen was studied by CLSM. The depth of permeation inside the intestinal lumen for drug suspension was found to be 15 µm, while SNEDDS showed penetration up to 30 µm (Figure 8).

The extent of the permeation of SNEDDS into the intestinal wall depends on its permeation ability into the mucus layer. The mechanism by which SNEDDS can penetrate into the mucus membrane significantly depends on the surfactant. The entangled network of the mucin fiber provides a steric barrier and limits the permeation of the nanoparticle droplet in mucus. The surfactant is the only component present in the formulation that can alter the permeation ability into the mucus by decreasing the binding strength, altering the mucus structure and by enhancing the hydrophilicity of microenvironment. The interaction between the surfactant present on the surface of the droplet and mucin leads to the changes in the mucin confirmation, reduces the interaction between mucin fibers and increases the pore size of the mucus, which can facilitate the permeation of SNEDDS into the mucus [83]. The permeation into the intestine is in proportion to mucus permeation ability of the SNEDDS. It has been reported in the literature that the surfactant with an HLB value ranging from 10 to 17 enhanced the drug absorption and intestinal penetration by inhibiting the efflux pump, which might be due to the penetration and adsorption of the surfactant molecule on the surface of the efflux pump and disorienting the intestinal membrane. In this study, the permeation of SNEDDS increased in comparison to the suspension due to the presence of the surfactant Tween 80 (with HLB 15) in the SNEDDS [84]. Moreover, the droplet size in the nanometric range (<200 nm) could also be the reason for enhanced permeation as the smaller droplet can easily permeate via the intestinal wall. Similar findings of the permeation of a lipid-based formulation is reported in the established literature [29].

#### 3.7.5. Lipolysis by pH Stat Method

The in vitro lipolysis digestion models are widely used to study the influence of lipid-based formulation excipients on the lipid digestion in the in vitro environment that mimic the conditions of the small intestine. When lipids are present in the formulation, the gall bladder tries to squeeze out the endogenous biliary lipids, phospholipids, bile salts and cholesterol, converting the extracellular lipids into monoglycerides, fatty acids and lysophospholipids, ultimately resulting in a variety of colloidal structures, such as micelles, unilamellar and multilamellar vesicles. These colloidal structures developed from the formulation deliver the digested lipid along with drugs to the aqueous enterocytes interface for the maximum absorption of the drug [67]. Various studies reported that the rate of lipolysis is inversely proportional to the droplet size of oil globules. As the droplet size decreases, the interfacial area increases with enhanced drug absorption via lumen of the intestine [85]. After oral administration, the drug should be dissolved in the intestinal tract for the maximum absorption to produce enhanced bioavailability. In the present work, optimized SNEDDS containing capmul MCM, a medium-chain triglyceride, was fully digested during lipolysis and was converted into FFA during the aqueous phase in the presence of various digestive enzymes between 15-20 min (Figure 7c). The separated aqueous and sediment phase obtained after the completion of lipolysis was quantified for the concentration of drugs by HPLC. After the lipolysis process, the concentration of TAM and RES were found to be 91.01 ± 1.00% and 94.06 ± 1.10%, respectively, in the aqueous phase, which is available for absorption in the lumen of the intestine. The aqueous phase showed a high concentration of the drugs (TAM and RES) compared to the sediment phase.

#### 3.7.6. Hemolysis Test

The morphological changes in RBCs after treatment with formulation were observed and recorded. The shape of normal RBCs is biconcave disc but, after exposing to some external agents, these shapes can get transformed into other shapes [86]. In the presence of suspension of drugs i.e., TAM and RES and triton X 100, the cells took the shape of spherocytes (Figure 7dA,B), whereas in the presence of PBS, placebo and SNEDDS, the shape of RBCs did not change (Figure 7dC–E). This study indicated that the pure drug deformed the RBCs as there was a change in the shape from a biconcave disc to spherocytes, whereas after the loading of the same concentration of drugs into the lipid-based formulation, no spherocytes were formed. The percentage of hemolysis observed with the SNEDDS and placebo were from approximately 2–3%, which lie in the safe zone, whereas the percentage of hemolysis caused by suspension was more than 11%. These results indicated that the drugs were toxic to RBCs, while the lipid-based formulation, such as SNEDDS, was safe and non-toxic to RBCs.

### 3.8. Cell Line Studies

#### 3.8.1. Cytotoxicity Studies

**Figure 9:**
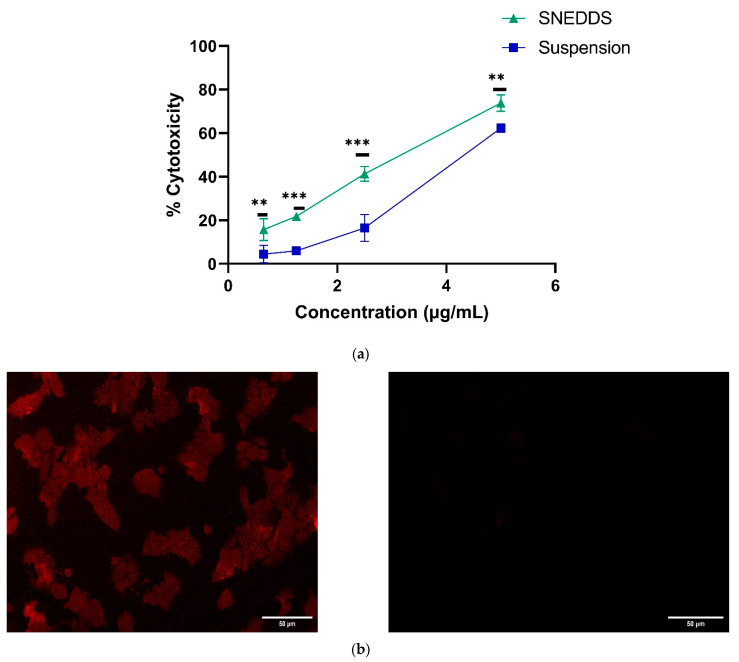

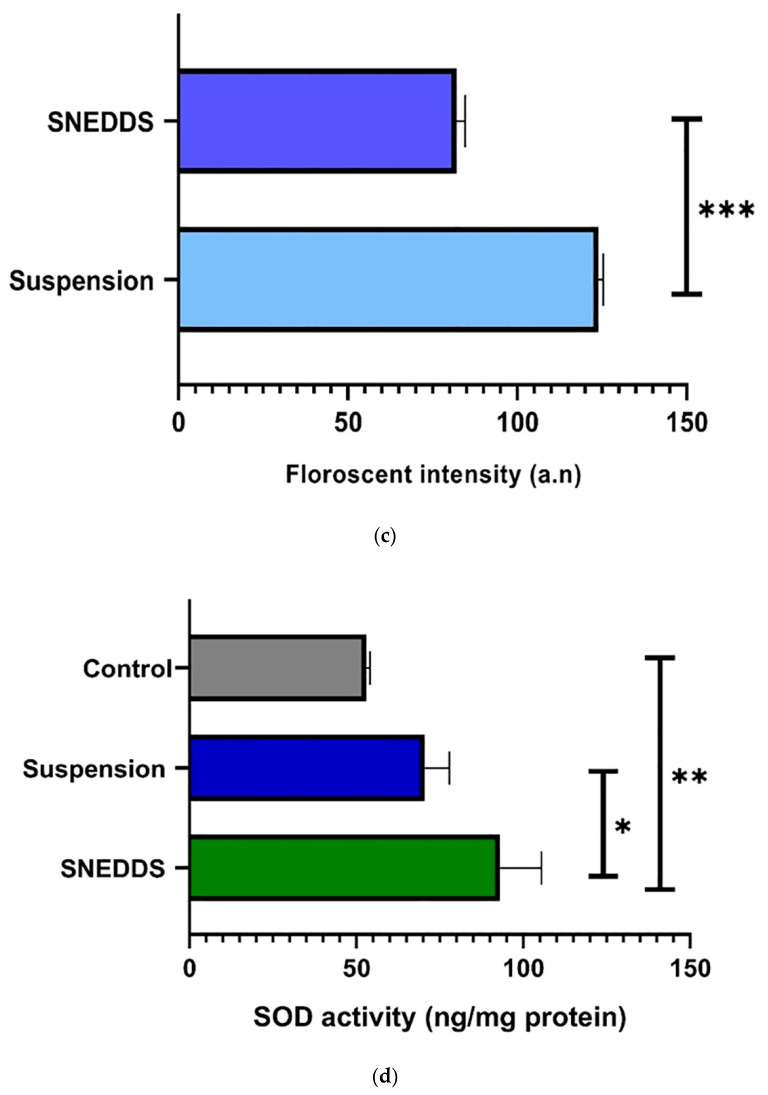
In vitro cell line study showed the (**a**) cytotoxicity of TAM-RES-s-SNEDDS and suspension after incubation with MCF-7 cell. (**b**) Fluorescent microscopic images of MCF-7 after incubation with A. SNEDDS, B. Suspension. (**c**) ROS level in MCF-7 after incubation with SNEDDS and suspension using DCFDA technique. (**d**) The SOD activity in MCF-7 after incubation with SNEDDS, suspension and control. Data are given as mean ± standard deviation (*n* = 3) with statistical difference at (*) *p* < 0.05, (**) *p* < 0.01 and (***) *p* < 0.001.

The in vitro cytotoxicity of TAM and RES-loaded SNEDDS and suspension was measured by determining the percentage of cytotoxicity against various concentrations of the formulations (Figure 9a). The IC_50_ of suspension was significantly higher (*p* < 0.05) than the SNEDDS, which was found to be 4.311 µg/mL and 3.224 µg/mL, respectively. The enhanced cytotoxicity of drugs from SNEDDS was due to the improved bioavailability of the drugs from the formulation due to enhanced solubility and small droplet size, which resulted in increased permeation into the wall of the cell. This resulted in the rapid release of the drug, which easily reached the cell organelles thereby exhibiting cytotoxicity. Akhtartavan also reported similar findings of enhanced cytotoxicity and reduced IC50 of docetaxel-loaded SNEDDS rather than the suspension on MCF-7 [87]

#### 3.8.2. Cellular Uptake Studies

The cellular uptake of fluorescent formulation and suspension was visualized by fluorescent microscope after 4 h of incubation. The confocal images (Figure 9b) indicated the intercellular localization of SNEDDS into the cell membrane rather than the suspension, signifying the uniform distribution of SNEDDS rather than that of the suspension. The obtained results suggested that dual drug-loaded SNEDDS were able to cross the cell membrane due to the small droplet size [88]. Xu and his co-worker also reported the enhanced cellular uptake of the lipid-based formulation in MCF-7 [89].

#### 3.8.3. Estimation of Intracellular Antioxidant

##### Reactive Oxygen Species (ROS) in MCF-7 Cells by DCFDA Assay

The intercellular ROS production was evaluated by measuring the florescence of the DCFDA dye. The ROS production in the MCF-7 cell line treated with SNEDDS was less in comparison to the cell line treated with suspension (Figure 9c). The results of this study indicated that the RES, an antioxidant loaded in the lipid-based formulation, suppressed more ROS as compared to the RES present in the suspension form. For decades, TAM, an antiestrogen drug, has been used for the treatment of breast cancer. It has been reported that TAM induces cell death by accumulating in the phospholipid bilayer where it reversibly inactivates protein kinase C (PKC) and releases ROS in the mitochondria of breast cancer cells [90]. Bekele et al., reported that long-term administration of TAM leads to the development of resistance, which might be due to the excess production of ROS in the cells, resulting in a redox stress state [91]. In the redox stress state, various elements such as transcription factor, Nrf-2 and antioxidant response elements (ARE) get accumulated and develop the state of resistance against chemotherapy [92,93]. For supporting the above statement, the resistance against TAM could be reversed by inhibiting the generation of mitochondrial ROS by silencing the SOD enzymes or by directly killing the ROS [94]. In contrast, the disturbance in the redox state may lead to the survival or spreading of cancerous cells. Therefore, to maintain the equilibrium of ROS with TAM therapy, the antioxidant, such as RES, must be included in the treatment regimen. The results were found to be in agreement with those obtained by Henidi and co-workers, where the antioxidant ameliorated the ROS level back to normal in response to doxorubicin treatment in MCF-7 [95].

##### Assessment of Superoxide Dismutase Activity (SOD)

The SOD found in the cytosol and mitochondria of the cell is the first line defense against the free radical damage. The SOD levels in cells treated with suspension and SNEDDS were found to be 70.51 ± 7.36 and 93.02 ± 12.47 ng/mg protein in comparison to the control, i.e., 53.05 ± 1.07 ng/mg protein. Further, the level of SOD was significantly (*p* < 0.05) higher in the cells treated with SNEDDS as compared to the suspension (Figure 9d), which might be due to the lipid-based formulation that easily penetrated into the cytosol and mitochondria of the cells.

### 3.9. Pharmacokinetic Study

**Figure 10:**
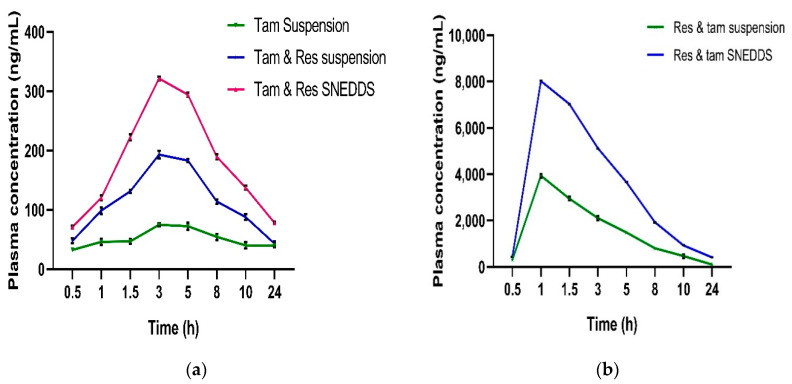
Plasma concentration time profile of (**a**) TAM and (**b**) RES after oral administration of their SNEDDS and suspension. Data expressed as mean ± SD, *n* = 6.

The pharmacokinetic study of TAM and RES was conducted on healthy Wistar rats for determining the bioavailability of both drugs released from suspension and SNEDDS in plasma. The peak plasma concentration (C_max_) and time required to reach (T_max_) was determined from the plot between plasma concentration of drugs versus time (Table 4; Figure 10).

The Cmax and AUC of TAM from TAM and RES suspension was enhanced 2.5 and 2.05 fold more than from the TAM suspension, respectively, indicating that the addition of RES added value to the formulation in terms of improved pharmacokinetics due to the inhibition of p glycoprotein efflux. The Cmax and AUC of TAM from SNEDDS significantly (*p* < 0.05) increased 1.66 and 1.63 folds more than the combination suspension, respectively. Further, the Cmax and AUC of TAM from combination SNEDDS significantly (*p* < 0.05) increased 4.16 and 3.35 fold more than from the TAM suspension. Poor pharmacokinetic profile of TAM is due to low solubility, high first pass metabolism and its susceptibility to the P-gp efflux pump inhibitor. Significantly better pharmacokinetic of TAM was observed after combining with RES and loading in a lipid-based formulation, which could be attributed to many reasons, the first being the small droplet size of nanoemulsion that could have easily entered into the lymphatic circulation and bypassed the first pass metabolism. Secondly, it could have been due to the presence of RES, which could prevent the efflux of TAM from the absorption site [96]. The results of pharmacokinetic studies are in accordance with the established literature as reported [97].

## 4. Conclusions

The TAM-RES-s-SNEDDS were formulated and characterized for various parameters. The promising variables, such as droplet size, PDI and percentage of transmittance, were optimized by response surface morphology via a central composite rotatable design. The ANOVA analysis demonstrated the effect of oil and S_mix_, which improved the SNEDDS properties. The in vitro release study showed that both drugs were entrapped in the nano droplets and released significantly higher than the conventional formulation. A non-everted gut sac study and CLSM were used to evaluate the formulation’s permeating effectiveness, which revealed a considerable improvement in drug permeation via SNEDDS as compared to the suspension. The cytotoxicity and internalization of SNEDDS in MCF-7 were significantly higher than in the suspension. The antioxidant potential of both drugs loaded SNEDDS was found to be higher than the suspension. The oral bioavailability of TAM (Cmax) from TAM-RES-s-SNEDDS was enhanced 4.16 folds more than from the TAM suspension. The above results show that TAM-RES-s-SNEDDS exhibit tremendous potential for delivering the drugs to the cancer cell via oral administration and prove that combinatorial therapy is a boon in the treatment of breast cancer.

## Figures and Tables

**Table 1:** Different level and constraints of variables.

Factors	Level Used				
Independent Variables	Axial (−α)	Lower (−1)	Medium (0)	Upper (+1)	Axial (+α)
Oil (mL) = X1	0.358579	0.4	0.5	0.6	0.641421
S_mix_ (mL) = X2	1.75858	1.8	1.9	2.00	2.04142
**Dependent Variables**	**Constraints**				
Droplet size (nm) = Y1	Minimum				
PDI = Y2	Minimum				
% Transmittance = Y3	Maximum				

**Table 2:** Effect of dilution on stability of formulation.

Dilutions	Formulations	Droplet Size (nm)	PDI	% Transmittance	Inference
50 folds	s-SNEDDS	87.98 ± 1.6	0.196 ± 0.21	97.41 ± 0.74	No phase separation
100 folds	s-SNEDDS	95.71 ± 3.29	0.368 ± 0.05	94.37 ± 0.21	No phase separation
200 folds	s-SNEDDS	95.75 ± 1.35	0.399 ± 0.47	94.57 ± 3.87	No phase separation

**Table 3:** Reconstitution behavior stability of s-SNEDDS in simulated gastrointestinal fluids.

Simulated Digestive Fluids	Droplet Size (nm)	PDI	% Transmittance	Inference
pH 1.2	72.78 ± 0.11	0.2 ± 0.003	91.62 ± 1.10	No phase separation
pH 6.8	78.33 ± 0.40	0.149 ± 0.004	92.87 ±0.80	No phase separation
**After incubation period**				
pH 1.2	82.94 ± 0.197	0.215 ± 0.002	90.307 ± 0.32	No phase separation
pH 6.8	84.46 ± 0.525	0.185 ± 0.011	94.275 ± 0.04	No phase separation

**Table 4:** Pharmacokinetic parameters.

Pharmacokinetic Parameters	TAM Suspension	TAM-RES-Suspension		TAM-RES-s-SNEDDS	
	TAM	TAM	RES	TAM	RES
C_max_ (ng/mL)	77.099	193.206	3933.471	321.480	8022.685
T_max_ (h)	3	3	1	3	1
AUC (ng/mL.h)	1113.495	2290.534	18,926.140	3737.314	44,533.762

Data are given as mean ± standard deviation (*n* = 6).

## Data Availability

Data is contained within the article.
